# Supplementary material for: A sand fly salivary protein acts as a neutrophil chemoattractant
Source: Nat Commun. 2021 May 28;12:3213. doi: 10.1038/s41467-021-23002-5 (PMC8163758; doi:10.1038/s41467-021-23002-5)
Supplement: Supplementary file 1 — Supplementary Information [file 41467_2021_23002_MOESM1_ESM.pdf]

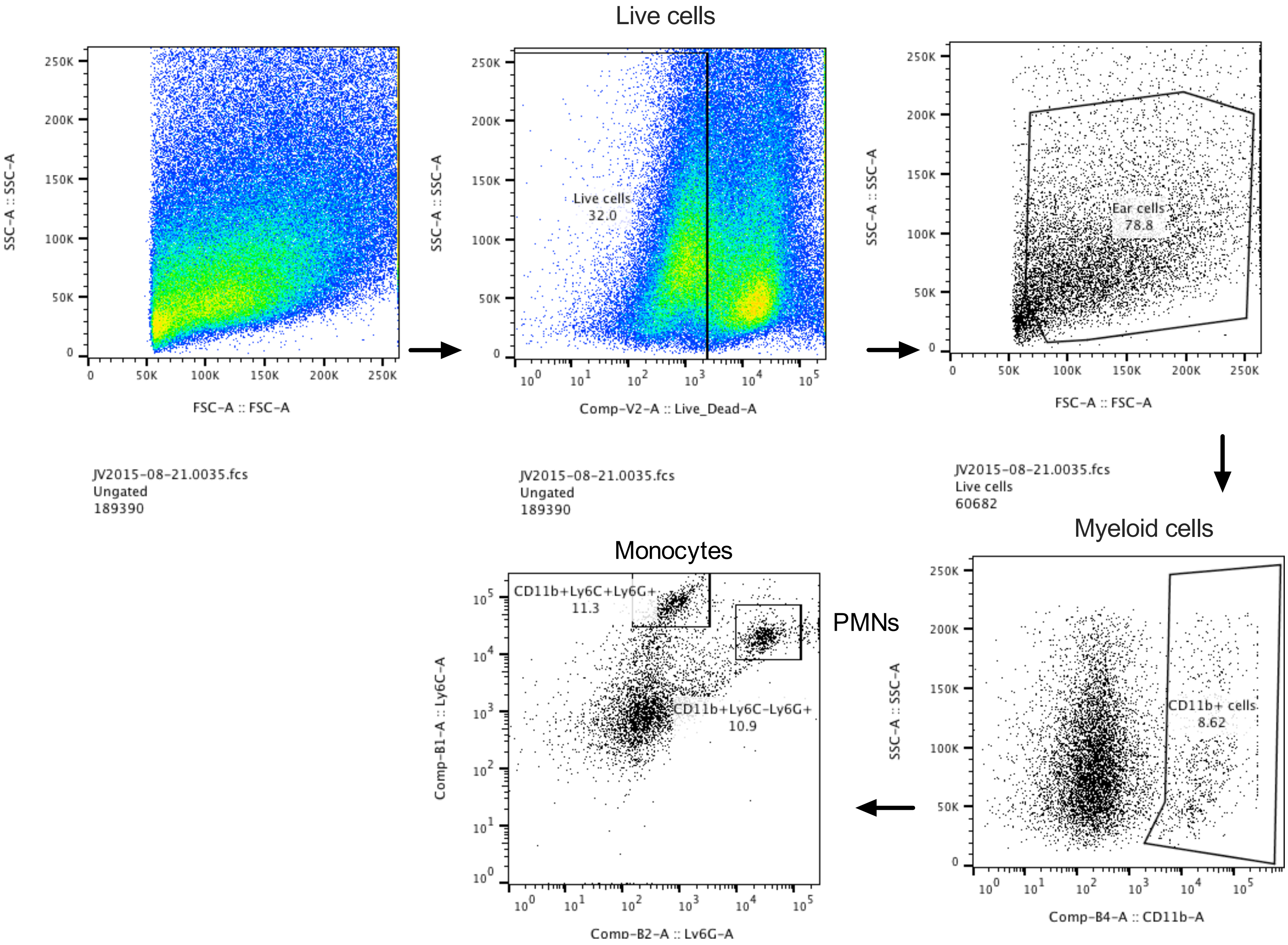

Supplementary Figure 1. Flow cytometry gating strategy.

Ear cell suspensions were labeled with fluorochrome-conjugated antibodies against surface markers of interest. The flow cytometry dot plots were obtained by gating on Live cells, CD11b+ cells (myeloid cells), followed by selection of neutrophils expressing Ly6G^{hi}, Ly6C^{hi} surface markers.

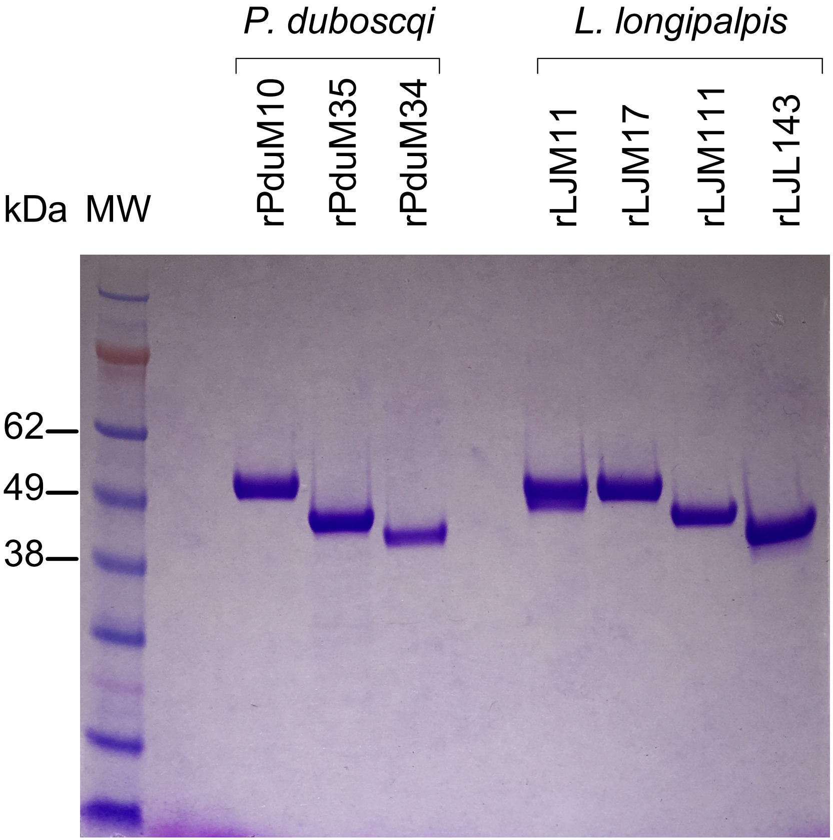

Supplementary figure 2. Recombinant proteins used in the study.

Recombinant proteins (200 ng each) from *P. duboscqi* (PduM10, PduM35 and PduM34) and *L. longipalpis* (LJM11, LJM17 and LJM111) were loaded into a NuPAGE 4-12% Bis-Tris gel under reducing conditions, followed by Coomassie brilliant blue staining. Representative of at least 3 experiments, recombinant proteins were run routinely before experiments to assay quality.

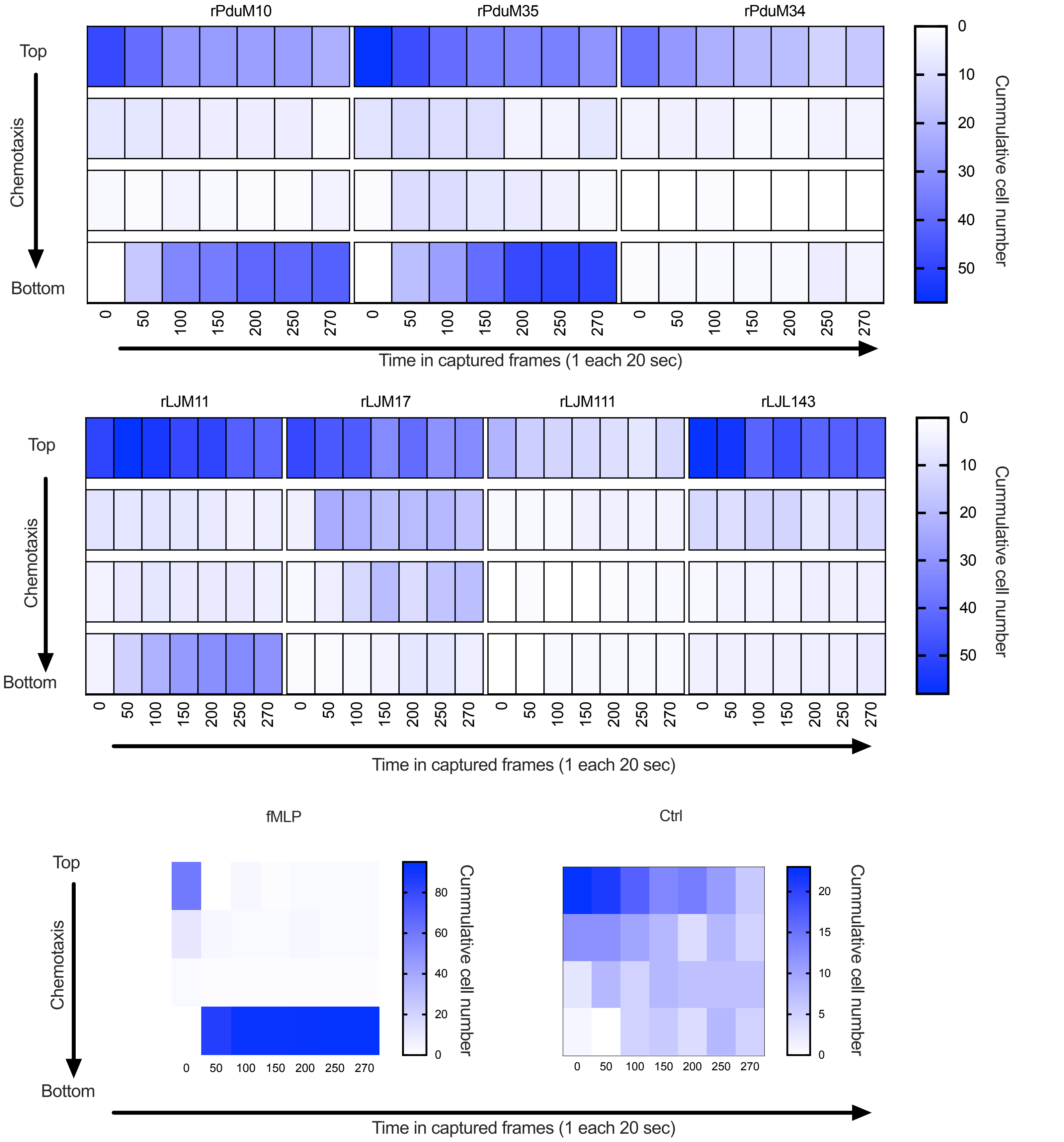

Supplementary figure 3. EZ-TAXIScan quantitative analysis of neutrophil migration towards salivary proteins. Heatmap shows the cumulative number of migrated neutrophils over captured frames to different stimuli. Each frame corresponds to 20 seconds of recorded neutrophil migration video.

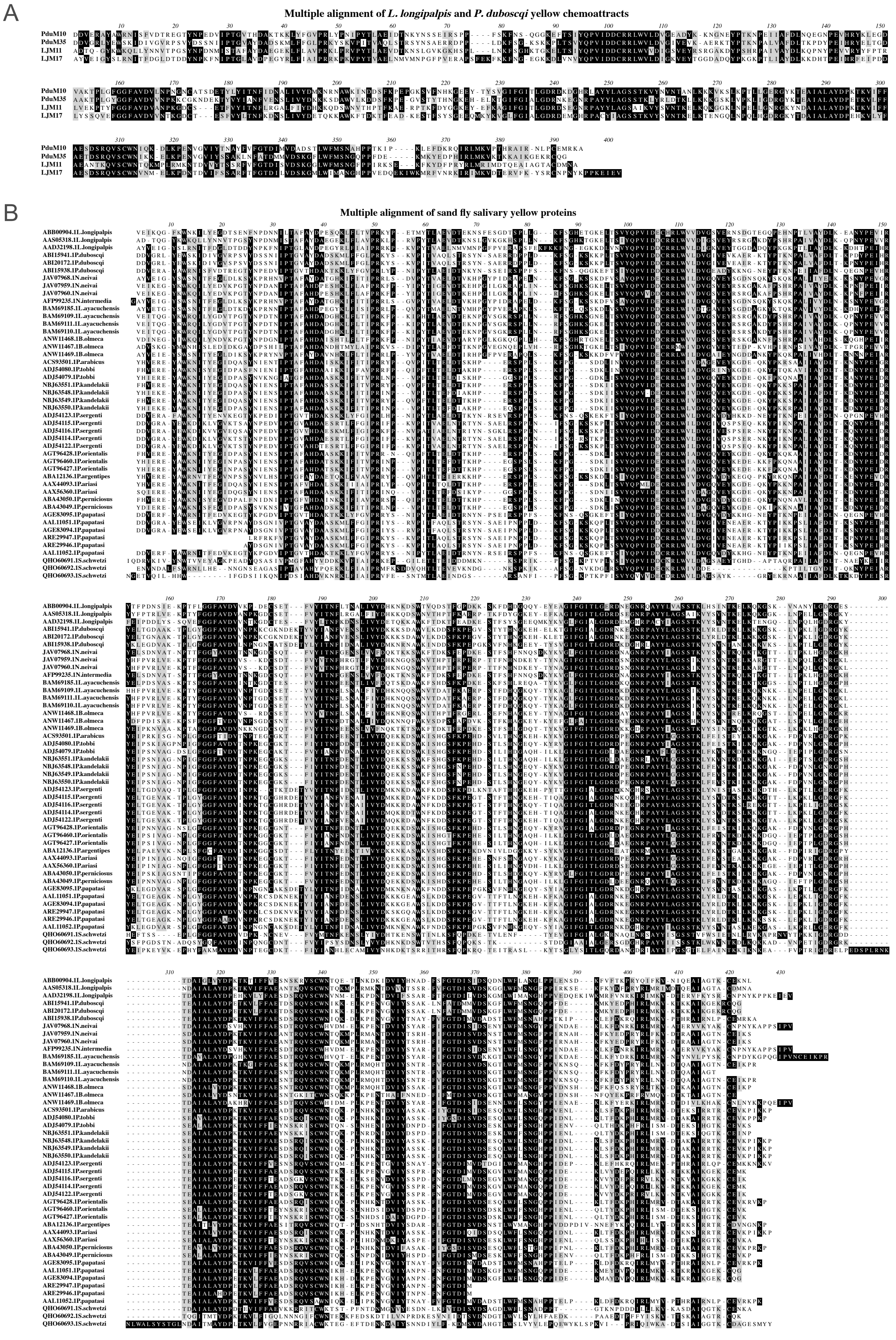

Supplementary figure 4. Identity and similarity among sand fly salivary yellow proteins

(A)Multiple alignments of *P. duboscqi* PduM10 and PduM35 with *L. longipalpis* LJM11 and LJM17. (B) Multiple alignments among available salivary Yellow-related proteins from sand flies using Muscle. Black shading represents identical amino acids, light gray shading represents similar amino acids.

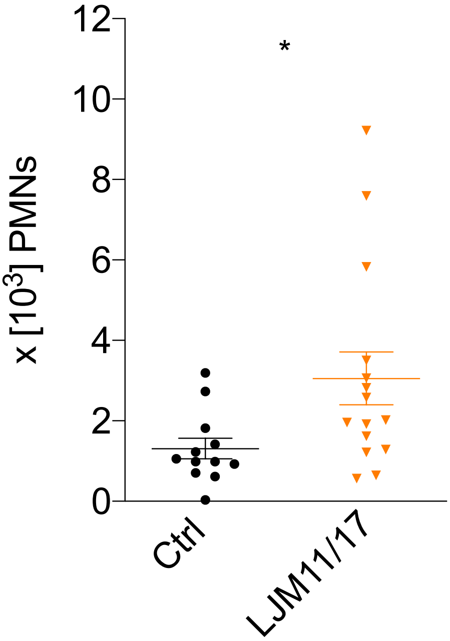

Supplementary figure 5. rLJM11 and rLJM17 act as chemoattractants for neutrophils in vivo.

Flow cytometry analysis of neutrophils recruitment to C57BL6 mice ears at 12 hours post-injection. Cell ears suspension was stained with anti-Ly6G, anti-Ly6C and CD11b antibodies. Cells Ly6G^{high}/CD11b^{+}/Ly6C$^{low/-}$ were considered as neutrophils. Mice ears were injected with PBS as negative control or LJM11 +LJM17 combination (250 nM). Results are shown as mean +/- SEM. Cumulative results of 2 independent experiments are presented as means +/- SEM; n = 12,14; *P =0.0214, calculated by Two-tailed Mann-Whitney test.

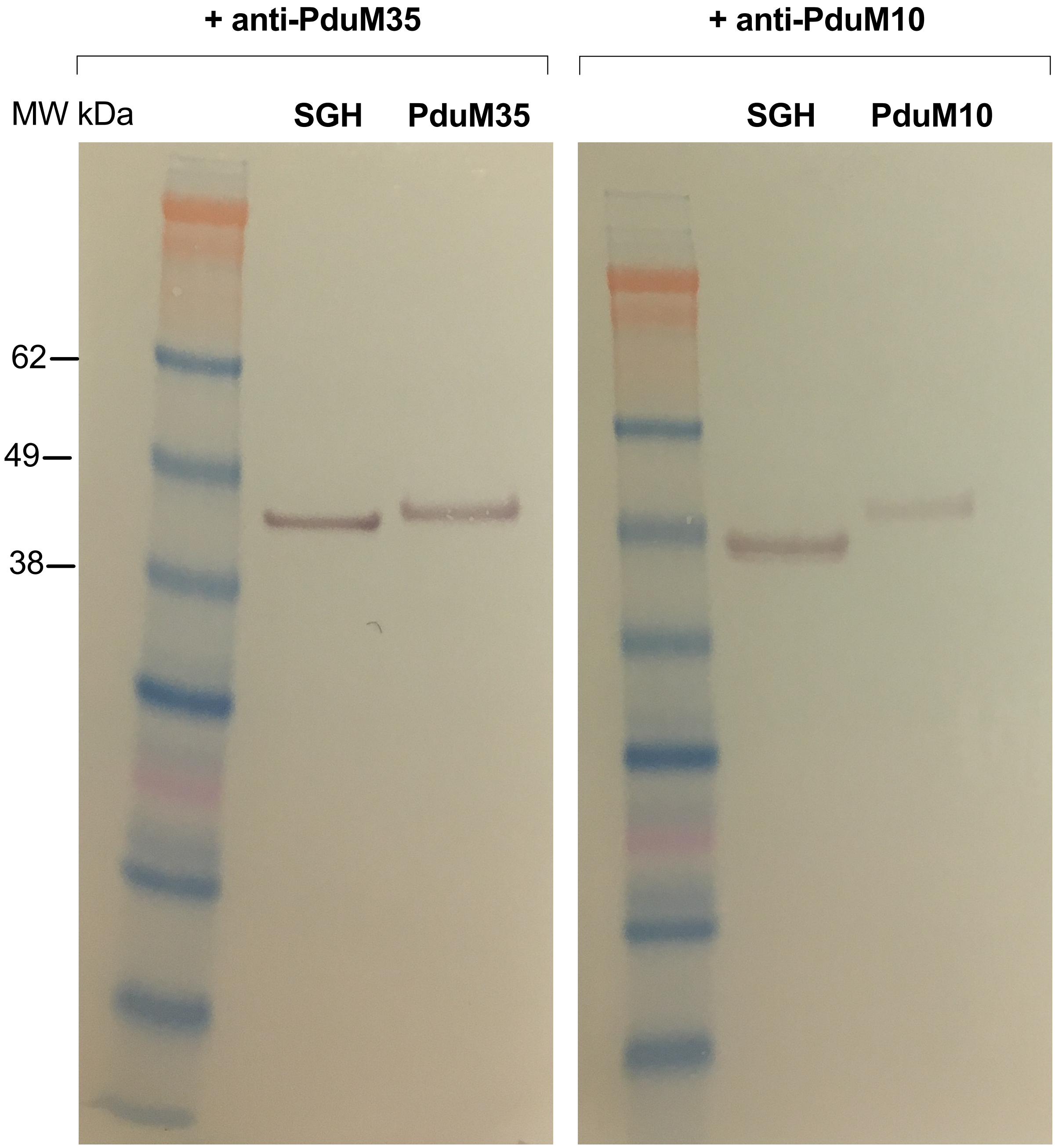

Supplementary figure 6. Antibodies anti-rPduM10 and rPduM35 specifically recognize the Yellow proteins in SGH.

P. duboscqi SGH (5 pairs of salivary glands), 500 ng of rPduM35 (left) or rPduM10 (right) were loaded into a NuPAGE 4-12% Bis-Tris gel and transferred to a nitrocellulose membrane, followed by incubation with anti-rPduM10 and anti-rPduM35 purified IgGs and secondary Alkaline Phosphatase-conjugated anti-mouse IgG. Both antibodies recognize a specific band in *P. duboscqi* SGH. Representative of at least 3 experiments, western-blots were run routinely before experiments to assay antibody quality overtime.

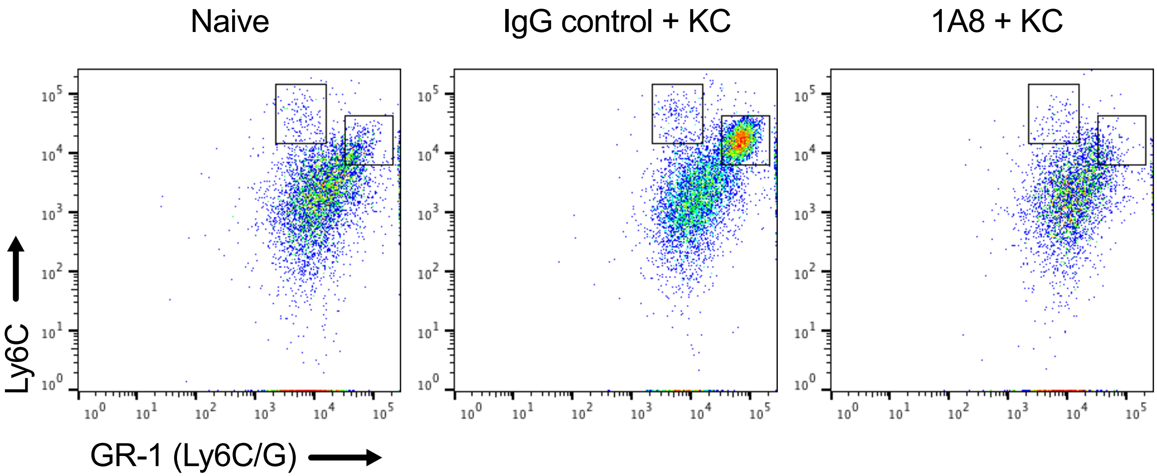

Supplementary Figure 7. Neutrophil Depletion

Representative flow cytometry dot plots showing the depletion of neutrophils (CD11b+Ly6C^{hi}Ly6G^{hi}) after 1A8 antibody administration.
