# Supplementary material for: A sand fly salivary protein acts as a neutrophil chemoattractant
Source: Nat Commun. 2021 May 28;12:3213. doi: 10.1038/s41467-021-23002-5 (PMC8163758; doi:10.1038/s41467-021-23002-5)
Supplement: Supplementary file 3 — Description of Additional Supplementary Files [file 41467_2021_23002_MOESM3_ESM.pdf]

Description of Additional Supplementary Files

Supplementary Movie 1: Neutrophil migration in response to 10 µg of *P. duboscqi* salivary gland homogenate (SGH) using an EZ-TAXIScan assay.

Supplementary Movie 2: Neutrophil migration in response to fMLP, a positive control, using an EZ-TAXIScan assay.

Supplementary Movie 3: Neutrophil migration in response to RPMI/0.1% BSA, a negative control, using an EZ-TAXIScan assay.

Supplementary Movie 4: Neutrophil migration in response to 100nM of rPduM10 using an EZ-TAXIScan assay.

Supplementary Movie 5: Neutrophil migration in response to fMLP, a positive control, using an EZ-TAXIScan assay.

Supplementary Movie 6: Neutrophil migration in response to RPMI/0.1% BSA, a negative control, using an EZ-TAXIScan assay.

Supplementary Movie 7: Intravital multiphoton imaging of neutrophil migration in eGFP-LysM C57BL/6 mice ears. Mice ears were injected with a negative control (PBS), 0.25 µM of each of rPduM10 + rPduM35 (isolated salivary proteins) or 500 ng of *P. dubosqci* salivary gland homogenate (sand fly saliva). Neutrophil (green) migration was recorded overtime. Collagen is stained in blue.
