# Supplementary material for: A sand fly salivary protein acts as a neutrophil chemoattractant
Source: Nat Commun. 2021 May 28;12:3213. doi: 10.1038/s41467-021-23002-5 (PMC8163758; doi:10.1038/s41467-021-23002-5)
Supplement: Supplementary file 11 — Reporting Summary [file 41467_2021_23002_MOESM11_ESM.pdf]

nature research

Corresponding author(s): Fabiano Oliveira and Jesus G Valenzuela

Last updated by author(s): March 2nd, 2021

# Reporting Summary

Nature Research wishes to improve the reproducibility of the work that we publish. This form provides structure for consistency and transparency in reporting. For further information on Nature Research policies, see our Editorial Policies and the Editorial Policy Checklist.

## Statistics

For all statistical analyses, confirm that the following items are present in the figure legend, table legend, main text, or Methods section.

| n/a | Confirmed | |
|---|---|---|
| ☐ | ☒ | The exact sample size (*n*) for each experimental group/condition, given as a discrete number and unit of measurement |
| ☐ | ☒ | A statement on whether measurements were taken from distinct samples or whether the same sample was measured repeatedly |
| ☐ | ☒ | The statistical test(s) used AND whether they are one- or two-sided<br>*Only common tests should be described solely by name; describe more complex techniques in the Methods section.* |
| ☒ | ☐ | A description of all covariates tested |
| ☒ | ☐ | A description of any assumptions or corrections, such as tests of normality and adjustment for multiple comparisons |
| ☐ | ☒ | A full description of the statistical parameters including central tendency (e.g. means) or other basic estimates (e.g. regression coefficient) AND variation (e.g. standard deviation) or associated estimates of uncertainty (e.g. confidence intervals) |
| ☐ | ☒ | For null hypothesis testing, the test statistic (e.g. *F*, *t*, *r*) with confidence intervals, effect sizes, degrees of freedom and *P* value noted<br>*Give P values as exact values whenever suitable.* |
| ☒ | ☐ | For Bayesian analysis, information on the choice of priors and Markov chain Monte Carlo settings |
| ☒ | ☐ | For hierarchical and complex designs, identification of the appropriate level for tests and full reporting of outcomes |
| ☒ | ☐ | Estimates of effect sizes (e.g. Cohen's *d*, Pearson's *r*), indicating how they were calculated |

*Our web collection on statistics for biologists contains articles on many of the points above.*

## Software and code

Policy information about availability of computer code

| | |
|---|---|
| Data collection | Intravital data were acquired on a Leica DMi8 inverted 5-channel confocal microscope (Leica Microsystems) equipped with MaiTai and InSight DeepSea lasers (Spectra Physics). The mean fluorescent intensity of the GFP channel was calculated from MIPs using Fiji. In vitro neutrophil migration videos were recored on a EZ-Taxiscan equipment (Effector Cell Institute, Tokyo, Japan). Transwell assay data were obtained through migrated cell DNA content on a Spectramax Gemini XPS fluorescence microplate reader (Molecular Devices, Menlo Park, CA). |
| Data analysis | 1. Imaris software (VERSION9.2) was used to analyze neutrophil migration videos. The software is available at http://www.bitplane.com/Default.aspx .<br>2. Flow cytometry data were obtained on a MACSQuant flow cytometer (Miltenyi Biotec) and analyzed by FlowJo Software 4.3. https://www.flowjo.com/solutions/flowjo/downloads<br>3. GraphPad Prism version 8 for Mac was used for statistical analysis. The statistical tests used throughout the study are shown in figure legends. https://www.graphpad.com/scientific-software/prism/<br>4. FIJI (Image processing package of ImageJ) was used to quantifiy neutrophil GFP fluorescence -Available at https://imagej.net/Fiji/Downloads<br>5. Image J (Version 1.52) is available at https://imagej.nih.gov/ij/download.html<br>6. SignalP-5.0 server (Version 5) was used to predict signal peptides. It is available at http://www.cbs.dtu.dk/services/SignalP/<br>7. BLAST tool latest version is available at https://blast.ncbi.nlm.nih.gov/Blast.cgi<br>8. The NR database is available at https://ftp.ncbi.nlm.nih.gov/blast/db/ and TSA database can be found at https://www.ncbi.nlm.nih.gov/Traces/wgs/?view=TSA |

For manuscripts utilizing custom algorithms or software that are central to the research but not yet described in published literature, software must be made available to editors and reviewers. We strongly encourage code deposition in a community repository (e.g. GitHub). See the Nature Research guidelines for submitting code & software for further information.

## Data

Policy information about availability of data

All manuscripts must include a data availability statement. This statement should provide the following information, where applicable:

- Accession codes, unique identifiers, or web links for publicly available datasets
- A list of figures that have associated raw data
- A description of any restrictions on data availability

The data that support the findings of this study are available from the corresponding author upon reasonable request.

# Field-specific reporting

Please select the one below that is the best fit for your research. If you are not sure, read the appropriate sections before making your selection.

☒ Life sciences ☐ Behavioural & social sciences ☐ Ecological, evolutionary & environmental sciences

For a reference copy of the document with all sections, see nature.com/documents/nr-reporting-summary-flat.pdf

# Life sciences study design

All studies must disclose on these points even when the disclosure is negative.

| | |
|---|---|
| Sample size | No particular sample size calculation was performed. Samples size were chosen based on published studies on murine and human cells and were based on biological variation within groups, to ensure reproducibility. The sample size (n) can be found in the figure legends.<br>Peters et al., Science 2008; Gomes et al., J Invest Dermatol 2012; Chagas et al., Plos Pathogen 2014. |
| Data exclusions | No data were excluded in this study. |
| Replication | Experiments were repeated two to three times to ensure reproducibility. This is described in the Figure legends |
| Randomization | Cages with C57BL/6 4-6 week old female mice were randomly assigned to groups. Mice were equally divided into control and experimental groups. For experiments with human subjects, all cells were treated the same way, so randomization was not needed. |
| Blinding | Investigators were blinded to sample origin and only during analysis were samples linked to treatment groups. |

# Behavioural & social sciences study design

All studies must disclose on these points even when the disclosure is negative.

| | |
|---|---|
| Study description | *Briefly describe the study type including whether data are quantitative, qualitative, or mixed-methods (e.g. qualitative cross-sectional, quantitative experimental, mixed-methods case study).* |
| Research sample | *State the research sample (e.g. Harvard university undergraduates, villagers in rural India) and provide relevant demographic information (e.g. age, sex) and indicate whether the sample is representative. Provide a rationale for the study sample chosen. For studies involving existing datasets, please describe the dataset and source.* |
| Sampling strategy | *Describe the sampling procedure (e.g. random, snowball, stratified, convenience). Describe the statistical methods that were used to predetermine sample size OR if no sample-size calculation was performed, describe how sample sizes were chosen and provide a rationale for why these sample sizes are sufficient. For qualitative data, please indicate whether data saturation was considered, and what criteria were used to decide that no further sampling was needed.* |
| Data collection | *Provide details about the data collection procedure, including the instruments or devices used to record the data (e.g. pen and paper, computer, eye tracker, video or audio equipment) whether anyone was present besides the participant(s) and the researcher, and whether the researcher was blind to experimental condition and/or the study hypothesis during data collection.* |
| Timing | *Indicate the start and stop dates of data collection. If there is a gap between collection periods, state the dates for each sample cohort.* |
| Data exclusions | *If no data were excluded from the analyses, state so OR if data were excluded, provide the exact number of exclusions and the rationale behind them, indicating whether exclusion criteria were pre-established.* |

| | |
|---|---|
| Non-participation | *State how many participants dropped out/declined participation and the reason(s) given OR provide response rate OR state that no participants dropped out/declined participation.* |
| Randomization | *If participants were not allocated into experimental groups, state so OR describe how participants were allocated to groups, and if allocation was not random, describe how covariates were controlled.* |

# Ecological, evolutionary & environmental sciences study design

All studies must disclose on these points even when the disclosure is negative.

| | |
|---|---|
| Study description | *Briefly describe the study. For quantitative data include treatment factors and interactions, design structure (e.g. factorial, nested, hierarchical), nature and number of experimental units and replicates.* |
| Research sample | *Describe the research sample (e.g. a group of tagged Passer domesticus, all Stenocereus thurberi within Organ Pipe Cactus National Monument), and provide a rationale for the sample choice. When relevant, describe the organism taxa, source, sex, age range and any manipulations. State what population the sample is meant to represent when applicable. For studies involving existing datasets, describe the data and its source.* |
| Sampling strategy | *Note the sampling procedure. Describe the statistical methods that were used to predetermine sample size OR if no sample-size calculation was performed, describe how sample sizes were chosen and provide a rationale for why these sample sizes are sufficient.* |
| Data collection | *Describe the data collection procedure, including who recorded the data and how.* |
| Timing and spatial scale | *Indicate the start and stop dates of data collection, noting the frequency and periodicity of sampling and providing a rationale for these choices. If there is a gap between collection periods, state the dates for each sample cohort. Specify the spatial scale from which the data are taken* |
| Data exclusions | *If no data were excluded from the analyses, state so OR if data were excluded, describe the exclusions and the rationale behind them, indicating whether exclusion criteria were pre-established.* |
| Reproducibility | *Describe the measures taken to verify the reproducibility of experimental findings. For each experiment, note whether any attempts to repeat the experiment failed OR state that all attempts to repeat the experiment were successful.* |
| Randomization | *Describe how samples/organisms/participants were allocated into groups. If allocation was not random, describe how covariates were controlled. If this is not relevant to your study, explain why.* |
| Blinding | *Describe the extent of blinding used during data acquisition and analysis. If blinding was not possible, describe why OR explain why blinding was not relevant to your study.* |

Did the study involve field work? ☐ Yes ☐ No

## Field work, collection and transport

| | |
|---|---|
| Field conditions | *Describe the study conditions for field work, providing relevant parameters (e.g. temperature, rainfall).* |
| Location | *State the location of the sampling or experiment, providing relevant parameters (e.g. latitude and longitude, elevation, water depth).* |
| Access & import/export | *Describe the efforts you have made to access habitats and to collect and import/export your samples in a responsible manner and in compliance with local, national and international laws, noting any permits that were obtained (give the name of the issuing authority, the date of issue, and any identifying information).* |
| Disturbance | *Describe any disturbance caused by the study and how it was minimized.* |

# Reporting for specific materials, systems and methods

We require information from authors about some types of materials, experimental systems and methods used in many studies. Here, indicate whether each material, system or method listed is relevant to your study. If you are not sure if a list item applies to your research, read the appropriate section before selecting a response.

## Materials & experimental systems

| n/a | Involved in the study |
| --- | --- |
| ☐ | ☒ Antibodies |
| ☐ | ☒ Eukaryotic cell lines |
| ☒ | ☐ Palaeontology and archaeology |
| ☐ | ☒ Animals and other organisms |
| ☐ | ☒ Human research participants |
| ☒ | ☐ Clinical data |
| ☒ | ☐ Dual use research of concern |

## Methods

| n/a | Involved in the study |
| --- | --- |
| ☒ | ☐ ChIP-seq |
| ☐ | ☒ Flow cytometry |
| ☒ | ☐ MRI-based neuroimaging |

# Antibodies

Antibodies used

1. Rat Anti-Mouse Ly-6C, BD Pharmingen™, Cat #553104, Clone AL-21, Lot #7067529
2. Rat Anti-Mouse Ly-6G, BD Pharmingen™, Cat #551461, Clone 1A8, Lot #45938
3. Rat Anti-Mouse CD11b, BD Pharmingen™, Cat #561098, Clone M1/70, Lot #31688
4. TruStain FcX™ Rat anti-mouse CD16/32) Antibody, BioLegend, Cat #101319, Clone 93, Lot #B276722
5. Alkaline phosphatase goat anti-mouse , Promega Cat #S372B , Polyclonal , Lot # 312817
6. Rat Anti-Mouse Ly-6G and Ly-6C (GR1), BD Pharmingen™, Cat #557661, Clone RB6-8C5, Lot # 6077709
7. Rat InVivoMAb anti-mouse Ly6G, BioXCell, Cat #BE0075-1, Clone 1A8, Lot # 5002/1013
8. anti-PduM10 and anti-PduM35 were produced in-house by intradermally immunizing mice with recombinant PduM35 and PduM10 as described in Methods.

Validation

The antibodies used for flow cytometry, in vivo depletion of neutrophils and western blot are widely used and well validated in the literature.
1. https://www.bdbiosciences.com/us/reagents/research/antibodies-buffers/immunology-reagents/anti-mouse-antibodies/cellsurface-antigens/fitc-rat-anti-mouse-ly-6c-al-21/p/553104
2. https://www.bdbiosciences.com/us/reagents/research/antibodies-buffers/immunology-reagents/anti-mouse-antibodies/cellsurface-antigens/pe-rat-anti-mouse-ly-6g-1a8/p/551461
3. https://www.bdbiosciences.com/us/applications/research/stem-cell-research/mesenchymal-stem-cell-markers-bone-marrow/mouse/negative-markers/pe-cy7-rat-anti-cd11b-m170/p/561098
4. https://www.biolegend.com/en-us/products/trustain-fcx-anti-mouse-cd16-32-antibody-5683
5. https://www.promega.com/products/protein-detection/primary-and-secondary-antibodies/anti-mouse-igg-h-and-l-apconjugate/?catNum=S3721
6. https://www.bdbiosciences.com/us/reagents/research/antibodies-buffers/immunology-reagents/anti-mouse-antibodies/cell-surface-antigens/apc-cy7-rat-anti-mouse-ly-6g-and-ly-6c-rb6-8c5/p/557661
7. https://bxcell.com/product/invivomab-anti-m-ly-6g/
8. Validation was performed by western blot analysis - Supplementary Figure 6.

# Eukaryotic cell lines

Policy information about cell lines

| | |
| --- | --- |
| Cell line source(s) | HEK-293F cells were comercially purchased from ThermoFisher Scientific. |
| Authentication | None of the cells were authenticated |
| Mycoplasma contamination | Tested for Mycoplasma prior to master cell bank |
| Commonly misidentified lines (See ICLAC register) | We have not used any commonly misidentified lines |

# Palaeontology and Archaeology

| | |
| --- | --- |
| Specimen provenance | *Provide provenance information for specimens and describe permits that were obtained for the work (including the name of the issuing authority, the date of issue, and any identifying information).* |
| Specimen deposition | *Indicate where the specimens have been deposited to permit free access by other researchers.* |
| Dating methods | *If new dates are provided, describe how they were obtained (e.g. collection, storage, sample pretreatment and measurement), where they were obtained (i.e. lab name), the calibration program and the protocol for quality assurance OR state that no new dates are provided.* |

☐ Tick this box to confirm that the raw and calibrated dates are available in the paper or in Supplementary Information.

| | |
|---|---|
| Ethics oversight | *Identify the organization(s) that approved or provided guidance on the study protocol, OR state that no ethical approval or guidance was required and explain why not.* |

Note that full information on the approval of the study protocol must also be provided in the manuscript.

## Animals and other organisms

Policy information about studies involving animals; ARRIVE guidelines recommended for reporting animal research

| | |
|---|---|
| Laboratory animals | C57BL/6 and Balbc 4-6 weeks old female mice were purchased from Charles Rivers (Wilmington, MA). Dog blood was obtained from the Division of Veterinary Research at the National Institutes of Health.<br>Phlebotomus duboscqi (Mali strain) and Lutzomyia longipalpis (Jacobina strain) were reared at the Laboratory of Malaria and Vector Research, NIAID, NIH. Salivary glands were dissected from 5-7 old females. |
| Wild animals | The study did not involve wild animals |
| Field-collected samples | The study did not involve field-collected samples. |
| Ethics oversight | All animal experimental procedures were reviewed and approved by the National Institute of Allergy and Infectious Diseases (NIAID) Animal Care and Use Committee under animal study protocol LMVR-4e. |

Note that full information on the approval of the study protocol must also be provided in the manuscript.

## Human research participants

Policy information about studies involving human research participants

| | |
|---|---|
| Population characteristics | Donors must meet the eligibility criteria for volunteer whole blood donation defined in the Standards of the AABB2 and the Code<br>of Federal Regulations 21 CFR 640,3 as modified in the 2007 FDA/CBER guidance document on eligibility criteria for donors of human cells and cellular-based products,5 with the exception of foreign travel history and other disorders conditions as noted<br>below.<br>Ability of subject to understand and the willingness to sign the written informed consent document<br>Age greater than or equal to 18 years<br>Type: Participants currently recruited/enrolled<br>Gender: Male & Female<br>Min Age: 18<br>Max Age: 80 |
| Recruitment | Regular blood donors were part of the study if they were accepted for routine donation and provided informed consent, without further selection. All participants were healthy at the moment of sampling. Selection bias should be minimal since no clinical attributes are being tested in this study. |
| Ethics oversight | Public View: 99-CC-0168<br>Clinical Trials Number:<br>NCT00001846 |

Note that full information on the approval of the study protocol must also be provided in the manuscript.

## Clinical data

Policy information about clinical studies

All manuscripts should comply with the ICMJEguidelines for publication of clinical research and a completedCONSORT checklist must be included with all submissions.

| | |
|---|---|
| Clinical trial registration | *Provide the trial registration number from ClinicalTrials.gov or an equivalent agency.* |
| Study protocol | *Note where the full trial protocol can be accessed OR if not available, explain why.* |
| Data collection | *Describe the settings and locales of data collection, noting the time periods of recruitment and data collection.* |
| Outcomes | *Describe how you pre-defined primary and secondary outcome measures and how you assessed these measures.* |

# Dual use research of concern

Policy information about dual use research of concern

## Hazards

Could the accidental, deliberate or reckless misuse of agents or technologies generated in the work, or the application of information presented in the manuscript, pose a threat to:

| No | Yes | |
|---|---|---|
| ☐ | ☐ | Public health |
| ☐ | ☐ | National security |
| ☐ | ☐ | Crops and/or livestock |
| ☐ | ☐ | Ecosystems |
| ☐ | ☐ | Any other significant area |

## Experiments of concern

Does the work involve any of these experiments of concern:

| No | Yes | |
|---|---|---|
| ☐ | ☐ | Demonstrate how to render a vaccine ineffective |
| ☐ | ☐ | Confer resistance to therapeutically useful antibiotics or antiviral agents |
| ☐ | ☐ | Enhance the virulence of a pathogen or render a nonpathogen virulent |
| ☐ | ☐ | Increase transmissibility of a pathogen |
| ☐ | ☐ | Alter the host range of a pathogen |
| ☐ | ☐ | Enable evasion of diagnostic/detection modalities |
| ☐ | ☐ | Enable the weaponization of a biological agent or toxin |
| ☐ | ☐ | Any other potentially harmful combination of experiments and agents |

# ChIP-seq

## Data deposition

☐ Confirm that both raw and final processed data have been deposited in a public database such as GEO.

☐ Confirm that you have deposited or provided access to graph files (e.g. BED files) for the called peaks.

| | |
|---|---|
| Data access links<br>*May remain private before publication.* | *For "Initial submission" or "Revised version" documents, provide reviewer access links. For your "Final submission" document, provide a link to the deposited data.* |
| Files in database submission | *Provide a list of all files available in the database submission.* |
| Genome browser session<br>(e.g. UCSC) | *Provide a link to an anonymized genome browser session for "Initial submission" and "Revised version" documents only, to enable peer review. Write "no longer applicable" for "Final submission" documents.* |

## Methodology

| | |
|---|---|
| Replicates | *Describe the experimental replicates, specifying number, type and replicate agreement.* |
| Sequencing depth | *Describe the sequencing depth for each experiment, providing the total number of reads, uniquely mapped reads, length of reads and whether they were paired- or single-end.* |
| Antibodies | *Describe the antibodies used for the ChIP-seq experiments; as applicable, provide supplier name, catalog number, clone name, and lot number.* |
| Peak calling parameters | *Specify the command line program and parameters used for read mapping and peak calling, including the ChIP, control and index files used.* |
| Data quality | *Describe the methods used to ensure data quality in full detail, including how many peaks are at FDR 5% and above 5-fold enrichment.* |
| Software | *Describe the software used to collect and analyze the ChIP-seq data. For custom code that has been deposited into a community repository, provide accession details.* |

# Flow Cytometry

## Plots

Confirm that:

- [x] The axis labels state the marker and fluorochrome used (e.g. CD4-FITC).
- [x] The axis scales are clearly visible. Include numbers along axes only for bottom left plot of group (a 'group' is an analysis of identical markers).
- [x] All plots are contour plots with outliers or pseudocolor plots.
- [x] A numerical value for number of cells or percentage (with statistics) is provided.

## Methodology

| | |
|---|---|
| Sample preparation | 1. Mice ears were collected and the two sheets of ear dermis were separated using sterile laboratory forceps<br>2. The two sheets of ear were deposited in PBS containing 0.2 mg/ml Liberase CI purified enzyme blend (Roche Diagnostics Corp)<br>and incubated for 1h at 37oC.<br>3. The digested tissue was placed in a grinder and processed in a tissue homogenizer Medimachine; Becton Dickenson<br>4. Tissue homogenates were filtered using a 30 µm Filcon filters (BD)<br>5. The resulting ear single cell suspensions were stained with Fixable Yellow Dead cell stain kit (Invitrogen) and then incubated<br>with anti-Fc (CD16/CD32).<br>6. Cells were then stained for Ly6C, Ly6G and CD11b for 30 min.<br>7. Cells were then washed three times and analyzed. |
| Instrument | MACSQuant flow cytometer (Miltenyi Biotec), (MACSquant 10) |
| Software | FLowjo v8.0 |
| Cell population abundance | no sorting was used in this study. |
| Gating strategy | 1. Cells were first gated on a FSC and SSC plot and then gated for singlets (FSC-H vs. FSCA).<br>2. Live cells were then gated based on the Yellow Fixable Dead Cell stain.<br>3. CD11b positive cells were gated to analyze the neutrophil population.<br>4. Inside the CD11b positive cells we analyzed the expression of Ly6C vs Ly6G.<br>Neutrophil is a very distinct population showing super bright staining for Ly6G. |

- [x] Tick this box to confirm that a figure exemplifying the gating strategy is provided in the Supplementary Information.

# Magnetic resonance imaging

## Experimental design

| | |
|---|---|
| Design type | *Indicate task or resting state; event-related or block design.* |
| Design specifications | *Specify the number of blocks, trials or experimental units per session and/or subject, and specify the length of each trial or block (if trials are blocked) and interval between trials.* |
| Behavioral performance measures | *State number and/or type of variables recorded (e.g. correct button press, response time) and what statistics were used to establish that the subjects were performing the task as expected (e.g. mean, range, and/or standard deviation across subjects).* |

## Acquisition

| | |
|---|---|
| Imaging type(s) | *Specify: functional, structural, diffusion, perfusion.* |
| Field strength | *Specify in Tesla* |
| Sequence & imaging parameters | *Specify the pulse sequence type (gradient echo, spin echo, etc.), imaging type (EPI, spiral, etc.), field of view, matrix size, slice thickness, orientation and TE/TR/flip angle.* |
| Area of acquisition | *State whether a whole brain scan was used OR define the area of acquisition, describing how the region was determined.* |

Diffusion MRI ☐ Used ☐ Not used

## Preprocessing

| | |
|---|---|
| Preprocessing software | *Provide detail on software version and revision number and on specific parameters (model/functions, brain extraction, segmentation, smoothing kernel size, etc.).* |
| Normalization | *If data were normalized/standardized, describe the approach(es): specify linear or non-linear and define image types used for transformation OR indicate that data were not normalized and explain rationale for lack of normalization.* |
| Normalization template | *Describe the template used for normalization/transformation, specifying subject space or group standardized space (e.g. original Talairach, MNI305, ICBM152) OR indicate that the data were not normalized.* |
| Noise and artifact removal | *Describe your procedure(s) for artifact and structured noise removal, specifying motion parameters, tissue signals and physiological signals (heart rate, respiration).* |
| Volume censoring | *Define your software and/or method and criteria for volume censoring, and state the extent of such censoring.* |

## Statistical modeling & inference

| | |
|---|---|
| Model type and settings | *Specify type (mass univariate, multivariate, RSA, predictive, etc.) and describe essential details of the model at the first and second levels (e.g. fixed, random or mixed effects; drift or auto-correlation).* |
| Effect(s) tested | *Define precise effect in terms of the task or stimulus conditions instead of psychological concepts and indicate whether ANOVA or factorial designs were used.* |

Specify type of analysis: ☐ Whole brain ☐ ROI-based ☐ Both

| | |
|---|---|
| Statistic type for inference (See Eklund et al. 2016) | *Specify voxel-wise or cluster-wise and report all relevant parameters for cluster-wise methods.* |
| Correction | *Describe the type of correction and how it is obtained for multiple comparisons (e.g. FWE, FDR, permutation or Monte Carlo).* |

## Models & analysis

| n/a | Involved in the study |
|---|---|
| ☐ | ☐ Functional and/or effective connectivity |
| ☐ | ☐ Graph analysis |
| ☐ | ☐ Multivariate modeling or predictive analysis |

| | |
|---|---|
| Functional and/or effective connectivity | *Report the measures of dependence used and the model details (e.g. Pearson correlation, partial correlation, mutual information).* |
| Graph analysis | *Report the dependent variable and connectivity measure, specifying weighted graph or binarized graph, subject- or group-level, and the global and/or node summaries used (e.g. clustering coefficient, efficiency, etc.).* |
| Multivariate modeling and predictive analysis | *Specify independent variables, features extraction and dimension reduction, model, training and evaluation metrics.* |
